# Multisystem Involvement in a Pediatric Patient With Suspected Mucopolysaccharidosis: A Case Report

**DOI:** 10.7759/cureus.60593

**Published:** 2024-05-19

**Authors:** Pratiksha Sachani, Rajasbala Dhande, Pratapsingh Parihar, Shivani S Bothara, Paschyanti R Kasat

**Affiliations:** 1 Radiodiagnosis, Jawaharlal Nehru Medical College, Datta Meghe Institute of Higher Education and Research, Wardha, IND

**Keywords:** growth hormone deficiency, metabolic disorder, pediatric patient, multisystem involvement, mri, mucopolysaccharidoses

## Abstract

Mucopolysaccharidoses (MPS) are a group of inherited metabolic disorders characterized by the deficiency or malfunction of lysosomal enzymes responsible for glycosaminoglycan (GAG) degradation. We present the case of an 11-year-old male with a history of calcified mitral valve, rheumatic heart disease, and growth hormone deficiency who presented with dyspnea on exertion. Physical examination revealed dysmorphic facial features, short stature, and suboptimal weight and height parameters. Magnetic resonance imaging (MRI) of the brain showed cystic lesions in the white matter and corpus callosum, hydrocephalus, and cerebral atrophy, suggestive of MPS. This case highlights the importance of considering MPS in the differential diagnosis of patients with multisystemic involvement and the utility of advanced imaging techniques like MRI in guiding diagnosis and management. A multidisciplinary approach involving cardiology, endocrinology, genetics, and neurology is crucial for comprehensive management and improving patient outcomes. Early diagnosis and intervention are essential in optimizing the quality of life for patients with MPS.

## Introduction

Mucopolysaccharidosis (MPS) encompasses a group of lysosomal storage disorders caused by the deficiency of specific enzymes required for glycosaminoglycan (GAG) degradation. These disorders are genetically inherited and lead to the accumulation of GAGs in cells, tissues, and organs, which manifests in various systemic symptoms and abnormalities [1]. The prevalence of MPS is estimated to be around one in 25,000 births, although this varies significantly across different types [2]. MPS clinical manifestations are diverse, depending on the specific type and severity of enzyme deficiency. They typically include organomegaly, dysostosis multiplex, and characteristic facial features. Neurological complications can occur, such as cognitive impairment, hydrocephalus, and spinal cord compression, reflecting the widespread impact of GAG accumulation [3]. Among the cardiovascular complications, valve disease is common, presenting most frequently as mitral and aortic valve dysfunction [4].

Rheumatic heart disease (RHD) continues to be a significant cause of mitral valve stenosis in regions where rheumatic fever is prevalent, and it complicates the clinical picture in patients with underlying conditions such as MPS [5]. Differentiating between RHD and MPS-related cardiac manifestations can be challenging but is crucial for appropriate management. Growth hormone deficiency (GHD) in MPS is not well-documented but may occur secondary to structural brain changes impacting the pituitary gland's function [6]. Growth hormone therapy, commonly used to address short stature in GHD, may have variable efficacy in MPS due to the underlying skeletal and systemic disease [7].

Advanced imaging techniques, particularly magnetic resonance imaging (MRI), are pivotal in diagnosing and monitoring MPS. MRI findings in MPS may include ventriculomegaly, enlarged perivascular spaces, and white matter lesions, which help distinguish MPS from other neurodegenerative conditions [8]. This case report aims to illustrate the complex interplay of symptoms, genetic factors, and multi-organ system involvement in a pediatric patient initially presenting with cardiac symptoms and subsequently diagnosed with a suspected metabolic disease like MPS based on a combination of clinical features and advanced imaging findings.

## Case presentation

**Figure 1:**
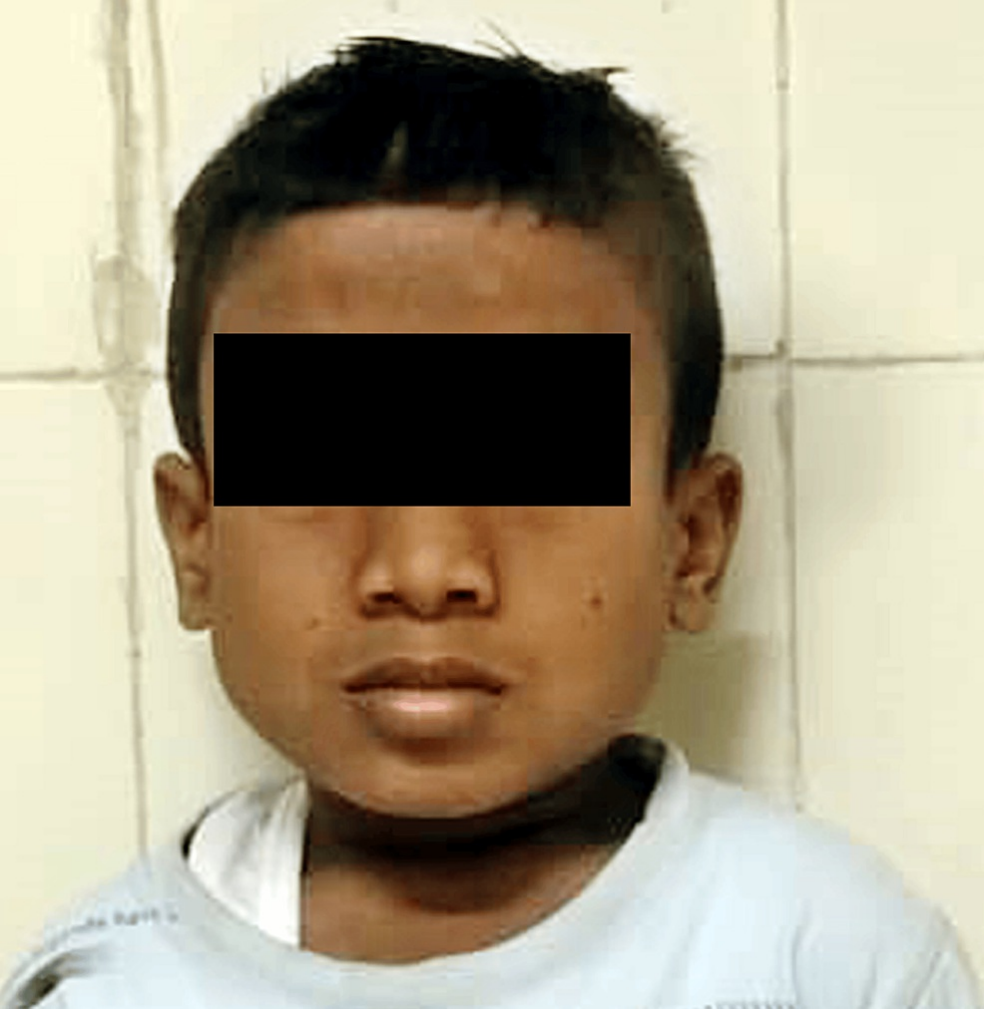
Physical examination of the patient showing a broad forehead, chubby cheeks, and a short neck

An 11-year-old male patient, known to have a calcified mitral valve leading to mitral valve stenosis and mild mitral regurgitation, as well as a history of RHD, was evaluated for ongoing health issues. He had been previously treated with penicillin and had a known GHD, for which he received growth hormone injections for three months, concluding in August 2023. The patient presented with a 1.5-year history of dyspnea on exertion but reported no current symptoms of fever, vomiting, or cough. He exhibited distinct facial features on physical examination, including a broad forehead, chubby cheeks, and a short neck (Figure 1). His weight and height were below the third percentile, and no significant birth history contributed to his current condition. His clinical presentation raised concerns about underlying systemic or metabolic disorders.

**Figure 2:**
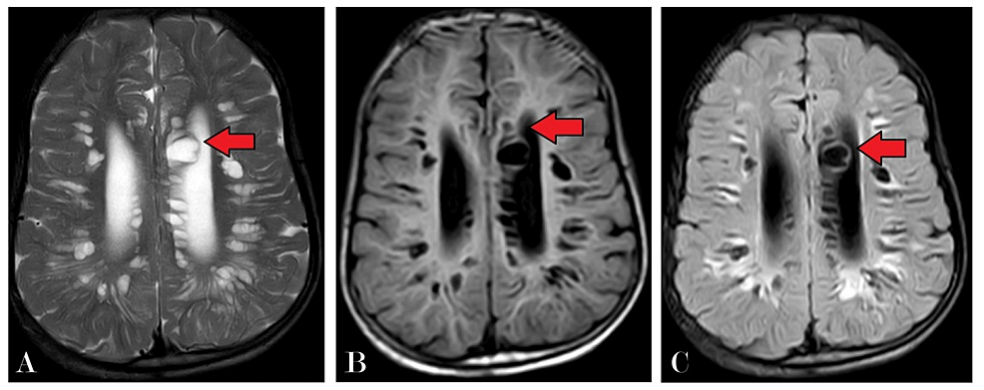
(A) T2WI, (B) T1WI, and (C) FLAIR: axial section of the brain showing multiple altered signal intensity areas mostly cystic, noted diffusely in the subcortical and deep periventricular white matter and corpus callosum appearing hyperintense on T2 and hypointense on T1 and FLAIR T2WI: T2-weighted imaging; T1WI: T1-weighted imaging; FLAIR: fluid-attenuated inversion recovery

**Figure 3:**
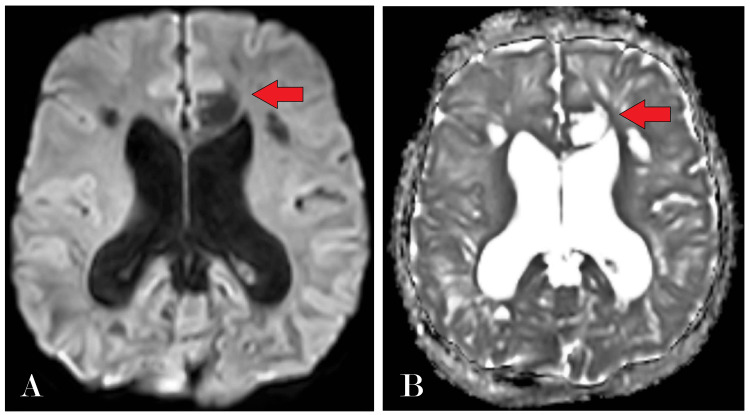
(A and B) Axial section of brain DWI and ADC sequence showing multiple altered signal intensity showing no diffusion restriction on DWI with corresponding low signal intensity on ADC DWI: diffusion-weighted imaging; ADC: apparent diffusion coefficient

**Figure 4:**
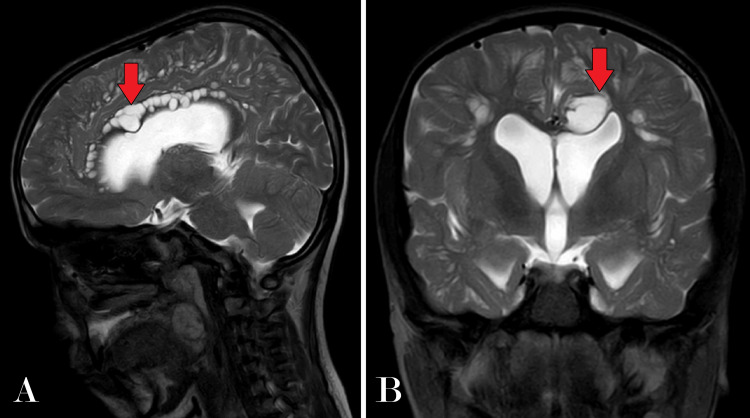
(A) Sagittal section of brain T2WI sequence showing multiple altered signal intensity areas mostly cystic, noted diffusely in the corpus callosum appearing hyperintense on T2WI. (B) Coronal section of brain T2WI showing a hyperintense cystic lesion in the body of the corpus callosum and dilatation of the ventricular system with an Evans index of 0.41 s/o ventriculomegaly T2WI: T2-weighted imaging

**Figure 5:**
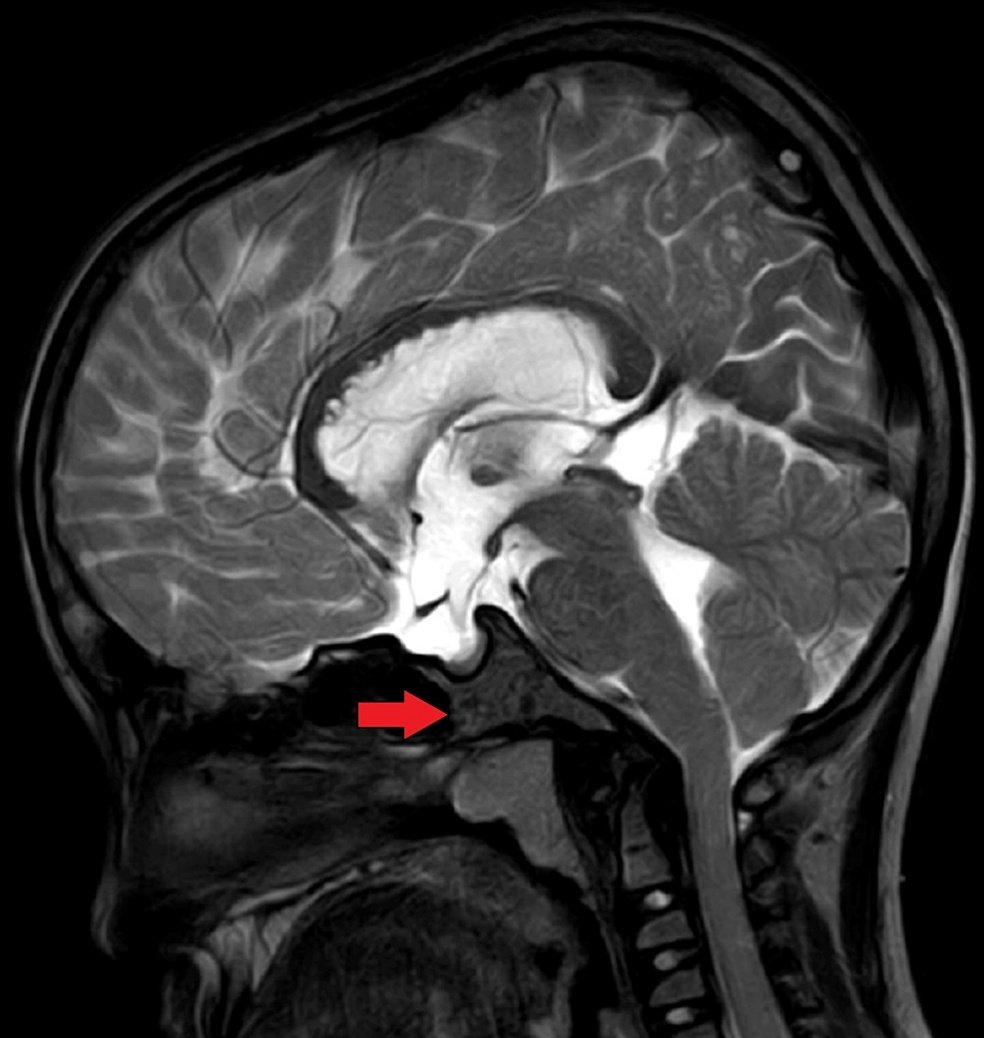
Sagittal section of brain T2WI sequence showing J-shaped sella turcica T2WI: T2-weighted imaging

Further diagnostic evaluation was pursued with an MRI of the brain, which revealed multiple cystic areas of altered signal intensity in the subcortical and deep periventricular white matter and the corpus callosum (Figure 2). These lesions appeared hyperintense on T2-weighted imaging (T2WI) and hypointense on T1-weighted imaging (T1WI) and fluid-attenuated inversion recovery (FLAIR) sequences, with no diffusion restriction (Figure 3), indicating the absence of acute infarcts. Notably, the MRI showed dilatation of all horns of the bilateral lateral ventricles and the third and fourth ventricles, suggesting hydrocephalus, quantified by an Evans index of 0.41 (Figure 4). There was also evidence of diffuse cerebral atrophy and a J-shaped sella turcica (Figure 5).

The constellation of findings from the MRI, coupled with the patient's clinical presentation and physical anomalies, suggested a possible diagnosis of a metabolic disease, likely MPS. This suspicion was based on the typical accumulation of GAGs affecting various tissues and organs, manifesting in both the skeletal abnormalities and the neurological imaging findings noted.

Given the complexity and multisystem involvement indicated by the initial assessments, a multidisciplinary approach involving cardiology, endocrinology, genetics, and neurology was recommended for comprehensive management. Genetic testing was proposed to confirm the diagnosis and to guide further treatment planning. Additionally, regular monitoring of cardiac function and neurodevelopment was advised, alongside supportive therapies aimed at managing the symptoms of hydrocephalus and the patient's GHD. The case highlighted the critical role of integrating detailed clinical evaluations with advanced diagnostic imaging to facilitate the diagnosis of complex, multi-faceted conditions like MPS.

## Discussion

MPS constitute a group of inherited metabolic disorders characterized by the deficiency or malfunction of lysosomal enzymes responsible for GAG degradation. As a result, GAGs accumulate in various tissues, leading to multisystemic manifestations [9]. The clinical presentation of MPS is heterogeneous and can involve the skeletal, cardiovascular, respiratory, and central nervous systems [10]. In the presented case, the patient exhibited characteristic clinical features such as short stature, dysmorphic facial features, cardiac abnormalities, and neurological symptoms, raising suspicion for an underlying metabolic disorder like MPS. The findings on MRI of the brain further supported this suspicion, demonstrating cystic lesions in the white matter and corpus callosum, along with hydrocephalus and cerebral atrophy. These neuroimaging features are consistent with those commonly observed in MPS, reflecting the widespread involvement of the central nervous system [11].

Cardiac involvement in MPS is well-documented, with valve abnormalities, cardiomyopathy, and conduction defects being common manifestations [12]. While the patient had a history of RHD and calcified mitral valve, these cardiac issues could be influenced by underlying MPS due to the deposition of GAGs in cardiac tissues [13]. The diagnosis of MPS typically relies on enzyme assays, genetic testing, and urine analysis of GAG levels [14]. However, in resource-limited settings or cases with ambiguous clinical presentations, advanced imaging techniques like MRI are crucial in suggesting the possibility of MPS and guiding further diagnostic workup [15]. Management of MPS involves a multidisciplinary approach aimed at addressing the diverse clinical manifestations and improving the quality of life. Enzyme replacement therapy and hematopoietic stem cell transplantation are available treatment modalities for certain types of MPS [16]. Additionally, supportive care targeting specific symptoms, such as cardiac, respiratory, and neurological complications, is essential in optimizing patient outcomes [17].

## Conclusions

This case highlights the importance of a comprehensive diagnostic approach integrating clinical assessment and advanced imaging techniques to guide timely diagnosis and appropriate management. Further confirmatory testing, such as enzyme assays or genetic analysis, is warranted to establish the diagnosis and initiate targeted therapies definitively. A multidisciplinary approach involving various specialists is essential for optimizing the care of patients with MPS, addressing the diverse systemic manifestations, and improving overall quality of life.
